# Left Ventricular Non-Compaction: Evolving Concepts

**DOI:** 10.3390/jcm13195674

**Published:** 2024-09-24

**Authors:** Raimondo Pittorru, Manuel De Lazzari, Federico Migliore, Enrica Frasson, Alessandro Zorzi, Alberto Cipriani, Giulia Brunetti, Giorgio De Conti, Raffaella Motta, Martina Perazzolo Marra, Domenico Corrado

**Affiliations:** 1 Department of Cardiac, Thoracic, Vascular Sciences and Public Health, University of Padova, 35128 Padua, Italy; raimondo.pittorru@studenti.unipd.it (R.P.);; 2 Radiology Unit, University of Padua-Azienda Ospedaliera, 35128 Padua, Italy

**Keywords:** left ventricular non-compaction, hypertrabeculation, cardiomyopathies, spongy myocardium

## Abstract

Left ventricular non-compaction (LVNC) is a rare heart muscle disease defined by the presence of prominent left ventricular trabeculation, deep intertrabecular recesses, and a thin compact layer. Several hypotheses have been proposed regarding its pathogenesis, with the most recently accepted one being that compact layer and trabeculated layers develop independently according to an “allometric growth”. The current gold-standard diagnostic criteria (in particular, the Petersen index non-compaction/compaction ratio > 2.3) reflect an excess of myocardial trabeculation, which is not a specific morpho-functional feature of LVNC cardiomyopathy but merely a “phenotypic trait”, even described in association with other myocardial disease and over-loading conditions. Accordingly, the European Society of Cardiology (ESC) guidelines have definitively abolished the term ‘LVNC cardiomyopathy’. Recently, evolving perspectives led to the restoration of LVNC cardiomyopathy by distinguishing “hypertrabeculation phenotype” and “non-compaction phenotype”. It has been proposed that the disease-specific pathophysiologic mechanism is a congenitally underdevelopment of the compact layer accounting for an impairment of the left ventricular systolic function. Future prospective research should focus on the clinical and prognostic relevance of compact layer thinning rather than excessive trabeculation, which could significantly influence the management of patients with LVNC. The review aims to update current knowledge on the pathogenesis, genetics, and diagnostic criteria of LVNC, offering modern insights for future perspectives.

## 1. Introduction

Left ventricular non-compaction (LVNC) is characterized by a prominent trabecular meshwork and extensive intertrabecular spaces that communicate directly with the ventricular cavity [1,2,3,4,5,6,7]. The myocardium in LVNC consists of two layers: a thicker, non-compacted layer containing ventricular cavities and interventricular recesses filled with blood and a thinner, subepicardial layer [3,4,5,6,7]. The real prevalence of LVNC is still unknown and highly variable according to the imaging modality used [2]. In adults, the prevalence is estimated to be about 0.5% [4]. Interestingly, the prevalence of LVNC with reduced left ventricular ejection fraction (LVEF) has been reported to be 3–5% [8,9]. Males and black patients are more frequently affected by LVNC compared to women and white patients [2,4,10].

LVNC has garnered increasing global recognition in recent years, drawing significant attention. Although various—often uncertain—etiologies may contribute to its development, LVNC has primarily been considered a congenital cardiomyopathy. Over time, the classification of LVNC has evolved, initially being categorized as an “unclassified cardiomyopathy” [11,12] and later as a “primary genetic cardiomyopathy” [1]. Arbustini et al. [13] suggested that LVNC represents a distinct phenotype, which may occur in isolation or alongside other cardiomyopathies, such as dilated cardiomyopathy (DCM) or hypertrophic cardiomyopathy (HCM) [14]. However, LVNC remains incompletely understood, and the presence of excessive trabeculation may overlap with other heterogeneous cardiac conditions. Moreover, the clinical significance of LVNC in adults is still not fully elucidated. Given the current gold-standard diagnostic criteria, accurately estimating the true incidence of LVNC is challenging. The condition encompasses a wide range of phenotypic presentations, including left ventricular dilation and dysfunction, and exhibits morphological changes that may oscillate between hypertrophic and dilated forms. In fact, the unresolved questions in LVNC concern the lack of universally accepted definition criteria and the unclear etiology. This review aims to summarize and update concepts on the pathogenesis, genetic basis, and diagnostic criteria of LVNC and provide modern insights for future research.

## 2. Congenital Etiology

The pathogenesis of left ventricular non-compaction (LVNC) has traditionally been attributed to an underdeveloped myocardial layer, thought to arise from an intrauterine arrest in the compaction process. This arrest results in the persistence of a loosely interwoven meshwork and deep trabecular recesses within the myocardial wall [7]. Normally, during embryonic development—specifically between five and eight weeks of gestation—the myocardium consists of a network of fibers with deep recesses. This trabeculation is essential for increasing surface area, thereby enabling adequate myocardial perfusion in the absence of coronary arteries. However, in LVNC, an interruption in the normal compaction process occurs from eight weeks of gestation onward due to disproportionate growth between the trabecular and compact layers [14,15,16,17,18,19,20,21]. This disruption prevents the trabeculae from coalescing into well-formed papillary muscles or from collapsing to form micro-circular capillary vessels, supporting the theory that LVNC is a congenital disorder. According to this view, LVNC represents the persistence of a trabecular network, a sponge-like muscle structure typical of mid- to late-embryonic life.

Recent studies, however, have challenged this traditional morphometric concept [22,23]. Emerging evidence suggests that the “allometric growth” theory of LVNC is outdated and not demonstrable [24]. Some research indicates that ventricular growth is a positive and continuous process without requiring the compaction of pre-existing trabeculation. Specifically, studies have shown that the growth of the compact layer occurs independently of the non-compact layer, as evidenced by experimental inhibition of trabecular proliferation and the induction of excessive trabeculation through the NKX2-5 pathway [25,26]. Further, a study by Rhee et al. [27] underscored the role of angiocrine factors in influencing cardiomyocyte behavior by modulating their proliferation and maturation. This emphasizes the critical interaction between endothelium and cardiomyocytes during the compaction process. These findings suggest a novel etiology for LVNC, where the dysregulation of paracrine signaling from endothelial cells might reduce cardiomyocyte proliferation, leading to a non-compaction phenotype [27].

## 3. Genetic Etiology

Several studies have explored the genetic background of LVNC. Initially, LVNC was classified as an inherited muscle disease with an autosomal dominant inheritance pattern [14,28,29]. However, it is now recognized that the genetic transmission of LVNC can also be autosomal recessive, X-linked, or mitochondrial, as reported in the literature [2,30]. Familial occurrences of LVNC have been documented and are considered a crucial factor in clinical assessment [31,32,33]. In a previous study, Hoedemaekers et al. [34] identified 11 pathogenic gene mutations, with myosin heavy chain being the most frequently observed defect, present in 17% of mutation carriers. There is a significant overlap in the genetic loci implicated in various cardiomyopathies, with sarcomeric proteins, particularly beta-myosin heavy chain, being the most commonly affected. Inherited molecular mechanisms or sporadic genetic mutations in cytoskeletal or sarcomeric proteins may lead to developmental anomalies in the myocardial layer, resulting in excessive trabeculation, which is a phenotypic trait shared by various forms of cardiomyopathies. To this regard, the most common mutations described are MYBPC3, TPM1, ACTC1, TNNT2, TNNI3, MYL2, MYL3, and MYH7 [18]. The latter, in patients with LVNC, is the most frequently documented sarcomeric gene involved [18]. Ion channels mutation such as SCN5A increase arrhythmia susceptibility in LVNC [35]. In addition, mutations in DMD (encoding-dystrophin) that cause Duchenne and Becker dystrophy have been implicated in LVNC [2,36]. Notably, the mutation of tafazzin results in Barth syndrome, which is typically characterized by LVNC [2]. Conversely, Ross et al. [37] conducted broad genetic testing on adult index patients with LVNC, suggesting that genetic testing is likely most beneficial in cases of LVNC associated with other cardiac features, such as reduced LVEF. They found it to be less useful in adults with isolated LVNC, especially in the absence of cardiac dysfunction or syndromic features [37]. Additionally, they advocate for the inclusion of transcription factors such as NKX2-5 in comprehensive gene panels, as these are primarily involved in the development of excessive trabeculation and advanced heart failure [26,37]. Concerning the understanding of the underlying mechanism of LVNC, other murine models have been studied [38]. For instance, disturbances in the NOTCH1 pathway such as FKBP1A-mediated regulation are crucial in controlling the formation of the ventricular walls [39]. Zhang W. et al. demonstrated that overexpression of TBX20 led to dilated cardiomyopathy (DCM) characterized by ventricular hypertrabeculation consistent with LVNC [38,40]. Remarkably, the dysregulation of Smad7 is associated with defects in cardiac development leading to LVNC with systolic dysfunction and arrhythmias [41]. It is noteworthy that LVNC does not have a mutation-specific correlation, and a strict genotype-phenotype relationship has not been established. The strongest genotype–phenotype correlations have been observed only for HCN4 and LMNA mutations [41,42]. These findings underscore the importance of conducting genetic investigations in at least the first-degree relatives of patients with LVNC.

## 4. Differential Diagnosis and Relevant Clinical Associations

Hypertrabeculation is not a distinctive morpho-functional marker for LVNC cardiomyopathy, for which diagnosis relies on critical thinning of the compact layer with systolic dysfunction (see the text below). The observation of hypertrabeculation patterns in adults has also led to the hypothesis that LVNC may be more of an acquired condition rather than strictly a congenital defect. Studies have documented that hypertrabeculation can appear as a phenotypic trait, particularly in athletes or pregnant women, as an adaptive response of myocardial architecture [17,43,44,45].

In particular, hypertrabeculation has been identified as a common phenotypic trait among trained athletes across various ethnicities and sports disciplines [29,46]. In fact, intensive physical activity, which imposes high demands on cardiac preload, can lead to the acquired development of prominent trabeculations [2,20,43,44,45]. Similarly, a non-negligible proportion of pregnant women with structurally normal hearts exhibit reversible secondary hypertrabeculation [44,45]. This adaptation is believed to result from hemodynamic overload, either transient or permanent, and may occur irrespective of genetic susceptibility [44,45]. Notably, both athletes following a period of detraining and women in the postpartum period exhibit complete regression of hypertrabeculation, suggesting that increased trabeculation allows for the same stroke volume to be generated with lower wall stress [43,44,45].

Hemoglobinopathies and other hematological disorders have also been linked to excessive trabeculation through similar pathophysiological mechanisms [46,47,48,49]. However, most individuals with hypertrabeculation maintain a preserved left ventricular ejection fraction, challenging the notion that hypertrabeculation is indicative of an underlying cardiomyopathy. The association between hypertrabeculation and neuromuscular disorders has been extensively studied. Neuromuscular disorders most frequently associated with LVNC include Barth syndrome, mitochondrial disorders, myotonic dystrophy, Holt-Oram syndrome, dystrobrevinopathy, and Emery–Dreifuss muscular dystrophy, particularly those involving LMNA mutations [2,33,36,50,51,52,53,54,55,56]. Despite the apparent connection, the actual proportion of patients with both neuromuscular disorders and LVNC is relatively low, suggesting that LVNC may manifest as a compensatory response in certain pathological contexts rather than as a direct consequence of these disorders.

LVNC has also been reported in association with various congenital heart diseases, including atrial septal defect, ventricular septal defect, bicuspid aortic valve, coronary artery anomalies, left ventricular outflow obstruction, tetralogy of Fallot, Ebstein’s anomaly, and patent ductus arteriosus [57,58]. As with neuromuscular disorders, the presence of hypertrabeculation in these cases may represent a compensatory process rather than an intrinsic component of the congenital defect. Additionally, the presence of a bilayered myocardium has been observed in conditions such as hypertrophic cardiomyopathy (HCM), hypertensive heart disease, and aortic stenosis [59].

## 5. Imaging-Based Definition

The gold-standard diagnostic criteria focus exclusively on the non-compacted layer, often neglecting the compacted layer [7,60,61,62,63]. The most widely used criteria are displayed in Table 1. This approach has led to an overdiagnosis of LVNC in a sizable proportion of asymptomatic and healthy individuals. In this context, cardiac magnetic resonance (CMR) imaging plays a crucial role, not only for diagnostic purposes but also for prognostic assessment, due to its high accuracy for definition of wall morphology. CMR provides better delineation between the non-compaction and compact myocardial layers and allows precise measurements of wall thickness. Moreover, CMR can be a valuable tool for arrhythmic risk stratification in these patients. Late gadolinium enhancement (LGE) on CMR, which indicates areas of myocardial fibrosis, has been reported in advanced diseases with significant LV dilatation/dysfunction. Although there is no specific LGE pattern that is pathognomonic for LVNC, Wan J. et al. [64] reported that the most common distribution of LGE in LVNC is midmyocardial, similarly to DCM.

## 6. Clinical Manifestation

LVNC presents a broad spectrum of clinical manifestations, including heart failure, arrhythmias, and thromboembolic events. The hypothesis that links left ventricular (LV) dysfunction directly to hypertrabeculation is inconsistent, as studies in human cohorts have shown only a weak correlation between hypertrabeculation and a decrease in ejection fraction. While LVNC predominantly affects the left ventricle, cases involving both ventricles or the right ventricle alone have also been reported in the literature [65].

Among the symptomatic triad of LVNC, arrhythmias can range from malignant ventricular tachyarrhythmias to supraventricular arrhythmias and atrial fibrillation. The absence of trabecular coalescence is often accompanied by alterations in the His–Purkinje fiber network, leading to a variety of conduction system abnormalities, such as paroxysmal supraventricular tachycardia, left or right bundle branch block, Wolff-Parkinson-White syndrome, atrioventricular block, early repolarization, and QTc prolongation [66,67]. Although LVNC has not been identified as the primary or sole cause of sudden cardiac death, it poses a significant concern, particularly for young people and athletes. Specific findings on ECG related to LVNC are sparse and mainly discussed in a few reviews. However, certain ECG patterns, particularly in trained athletes or black individuals, may raise suspicion of LVNC [68,69]. Changes such as T-wave inversion, ST-segment depression, pathological Q waves, and QRS fragmentation are more broadly indicative of cardiomyopathy. Therefore, it is essential to interpret the ECG with a specific “cardiomyopathy mindset”.

The unique architecture of LVNC, characterized by a meshwork of endocardial trabeculae and intertrabecular recesses, creates a substrate favorable for re-entrant ventricular arrhythmias (VAs). Additionally, the presence of left ventricular fibrosis increases the risk of VAs. However, the presence of myocardial fibrosis also raises questions about underlying structural heart disease, and some authors have excluded patients with LV scar to avoid confusion. Muser et al. demonstrated that both scar-related and focal VAs are present in LVNC, with a distinct pattern involving the LV mid-apical segments, setting it apart from other non-ischemic cardiomyopathies [70].

Thromboembolic events in LVNC are often due to thrombi that lodge in the deep recesses of the trabeculae. Case reports have described thromboembolic strokes associated with LVNC [71,72]. While hypertrabeculation and the Virchow triad suggest a higher incidence of intraventricular thrombosis, LV dysfunction remains the primary driver for clot formation.

## 7. Management and Treatment

The management of patients with LVNC follows a similar approach to that of other cardiomyopathies, including evidence-based heart failure therapy for LV systolic dysfunction, appropriate arrhythmia management, and consideration of oral anticoagulation to prevent thromboembolic events [73,74,75]. Currently, there are no specific therapeutic recommendations for LVNC codified in the ESC guidelines. The management of LVNC-related complications adheres to established protocols for the underlying conditions. For heart failure with systolic dysfunction, standard care involves the use of SGLT2 inhibitors, and in cases of end-stage heart failure that are refractory to optimal medical therapy, evaluation for a left ventricular assist device or heart transplantation is necessary. In cases of heart failure with diastolic dysfunction, the administration of SGLT2 inhibitors should be considered, in line with recent guidelines. However, in patients with concomitant neuromuscular disorders, it is important to consider the potential myotoxicity of immunosuppressive therapy. Al-Kindi et al. [76] highlighted that the overall outcomes for individuals undergoing heart transplantation were similar to those of patients with dilated cardiomyopathy. Takamatsu et al. reported a successful surgical resection of the non-compacted myocardial layer, resulting in improved EF. In terms of thromboembolic events, standard anticoagulant therapy should be administered following updated guidelines, with a focus on tailoring treatment to each patient’s thromboembolic risk. There are no specific guidelines for ICD implantation in LVNC patients. However, ICD implantation should be considered in patients with LVEF ≤ 35%, similar to recommendations for dilated cardiomyopathy [77]. Previous studies have shown that non-sustained ventricular tachycardia is the strongest indication for ICD implantation. Non-pharmacological treatments, such as cardiac resynchronization therapy (CRT), may also be beneficial. Bertini et al. [78] demonstrated reverse remodeling and EF improvement with cardiac resynchronization therapy (CRT). Additionally, a meta-analysis confirmed that CRT provides additional benefit in patients with heart failure and reduction of LVEF (≤35%), NYHA class 2–3, and left bundle branch block or QRS duration longer than 150 ms [79]. As demonstrated in the aforementioned studies, treatment with CRT led to a greater LV reverse remodeling in patients with LVNC compared to DCM [79]. Among LVNC patients, it was also observed a high percentage of super-responders to CRT compared to DCM population, especially when an LV epicardial lead paced the LVNC areas [78,79]. 

Sohns et al. [80] analyzed outcomes in LVNC patients who experienced multiple ventricular arrhythmias or recurrent ICD therapies, finding that both endocardial and endo-epicardial catheter ablation offered a safe and effective therapeutic option.

The risk of thromboembolic events in LVNC patients ranges from 15% to 38% [72]. There is no consensus on the optimal anticoagulation strategy for these patients, though anticoagulants are typically prescribed according to current guidelines. No studies have definitively determined whether vitamin K antagonists (VKAs) or direct oral anticoagulants (DOACs) are preferred, but VKAs may be more effective for endoventricular thrombosis, while DOACs may be better suited for patients with atrial fibrillation. In all cases, the risk–benefit ratio should be carefully evaluated based on each patient’s specific thromboembolic risk.

## 8. Prognosis

Specific and well-accepted recommendations for risk stratification in LVNC are currently lacking. However, the key prognostic factors are primarily driven by left ventricular ejection fraction (LVEF), ventricular arrhythmias, and thromboembolic events. Studies have suggested that increased trabeculation alone is associated with a favorable prognosis in both prospective and retrospective analyses [81,82,83]. A growing body of evidence, mainly from retrospective studies, indicates that hypertrabeculation is often an incidental finding in asymptomatic individuals with a low pretest probability of major cardiovascular events and a negative family history, resulting in a benign outcome [81,82,83]. In a meta-analysis conducted by Aung et al. [84], it was reported that reduced LVEF, rather than the extent of hypertrabeculation, is the primary determinant of prognosis in LVNC patients. Similarly, Grigoratos et al. [85] concluded that the amount of trabeculation does not predict major cardiovascular events. LVEF remains the most important and well-established factor associated with decompensated heart failure and major ventricular arrhythmias, consistent with other cardiomyopathies.

The presence of myocardial fibrosis, detected through late gadolinium enhancement (LGE), has been shown to significantly impact the risk of major ventricular arrhythmias. LGE has been consistently associated with increased risk of arrhythmias in various cardiomyopathies, even in the absence of LV systolic dysfunction [86]. The prognostic value of cardiovascular magnetic resonance (CMR) was further highlighted in a prospective multicenter study by Andreini et al. [87]. This study demonstrated that CMR can distinguish between patients at high risk of cardiovascular events and those with an excellent prognosis. Notably, patients with LV dilation and reduced LVEF had a worse prognosis compared to those without reduced LVEF and myocardial fibrosis. For instance, patients with LGE had a poor prognosis, regardless of LVEF status [87].

Casas et al. [88] developed a risk prediction model to guide the management of LVNC patients, although further external validation in larger cohorts is needed to ensure its clinical applicability. Overall, prognosis appears to be linked to a DCM-like phenotype with reduced LVEF, particularly when myocardial fibrosis is present. The main prognostic factors in LVNC patients are similar to those seen in DCM, but the natural history of the disease remains poorly understood due to confounding factors in patient enrollment.

Conversely, patients with isolated LVNC without LGE tend to have a good prognosis, similar to the general healthy population. In this context, athletes with isolated hypertrabeculation should not be restricted from training and competition [43].

## 9. European Society of Cardiology Statement

In 2023, the guidelines published by the Task Force of European Society of Cardiology (ESC) were clear in not considering LVNC a cardiomyopathy stricto sensu [75]. The authors definitively dismissed the term “cardiomyopathy” in favor of “hypertrabeculation”, drawing the conclusion that this is a phenotypic trait associated with other cardiomyopathies.

## 10. Left Ventricular Non-Compaction: A Paradigm Shift

**Figure 1:**
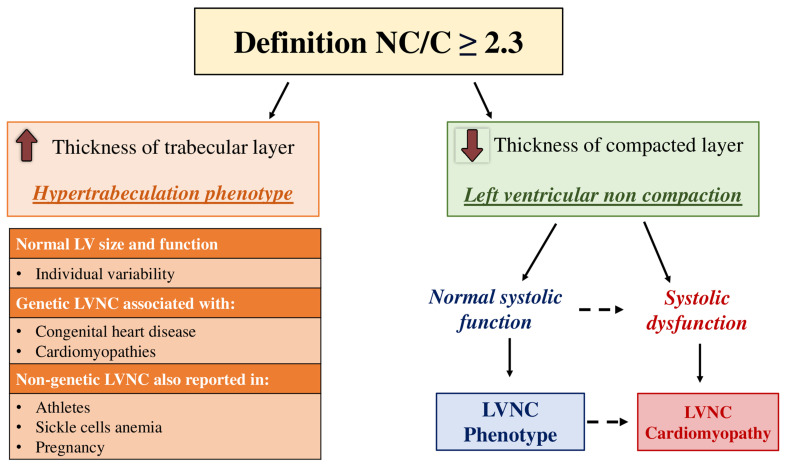
Graphical illustration. The NC/C ratio > 2.3 is due to an increase of numerator (non-compact layer) or a decrease of denominator (compact layer). An increased thickness of non-compact layer is the result of excessive trabeculation. This is a normal “phenotypic trait” observed in healthy individuals with normal LV size and function or a phenotypic feature superimposed on other heart muscle disease such as dilated cardiomyopathy and overloading conditions rather than a distinctive morpho-functional marker for LVNC cardiomyopathy. A reduction of thickness of the compact layer instead defines LVNC. Based on left ventricular ejection fraction (LVEF), we distinguish a “LVNC phenotype” characterized by preserved LVEF and “LVNC cardiomyopathy” characterized by reduction of LVEF. This implies that LVNC cardiomyopathy has a peculiar disease-specific mechanism. Legend: C = compact (layer); NC = non-compaction (layer); LV = left ventricle; LVNC = left ventricular non-compaction.

Cardiac magnetic resonance is considered the benchmark imaging technique for diagnosing left ventricular non-compaction. A widely accepted diagnostic criterion, introduced by Petersen et al., characterizes LVNC when the ratio of non-compacted to compacted myocardium at end-diastole exceeds 2.3 [60]. However, the Petersen index predominantly captures excessive trabeculation in the LV, a feature not exclusive to LVNC. Similar trabecular patterns are also found in other cardiac disorders as aforementioned above. Moreover, increased trabeculation can occur as a physiological variant in healthy individuals, particularly during pregnancy or after prolonged athletic activity, where it represents a reversible response to elevated ventricular load. These factors complicate the differentiation between “hypertrabeculation phenotype”, “non-compaction phenotype”, and “LVNC cardiomyopathy” [89] (Figure 1). Often, the presence of LV systolic dysfunction, alongside a positive Petersen index for excessive trabeculation, serves as a crucial marker for diagnosing true LVNC cardiomyopathy [89]. Yet, prior research has failed to establish a definitive link between the extent of trabeculation and impaired systolic function [81,85,87]. In response to these ambiguities, De Lazzari et al. proposed a “paradigm shift” concept of LVNC, focusing on the role of compact layer thinning in LVNC-associated systolic dysfunction [90]. Their case–control study compared patients meeting the Petersen LVNC criteria with LV dysfunction to a control group of age- and sex-matched individuals with LVNC but preserved systolic function [90]. The authors hypothesized that impaired systolic performance stems from underdevelopment of the compact layer rather than exaggerated trabeculation [90]. The final analysis showed that a compact layer thickness below 5 mm in the free-wall mid-ventricular segments was the most accurate predictor of systolic dysfunction in LVNC patients [90]. Specifically, having two or more segments with a compact layer thickness under 5 mm demonstrated 100% sensitivity and 60% specificity for reduced LVEF. Additionally, the absence of these features had a 100% negative predictive value for LV dysfunction [90].

**Figure 2:**
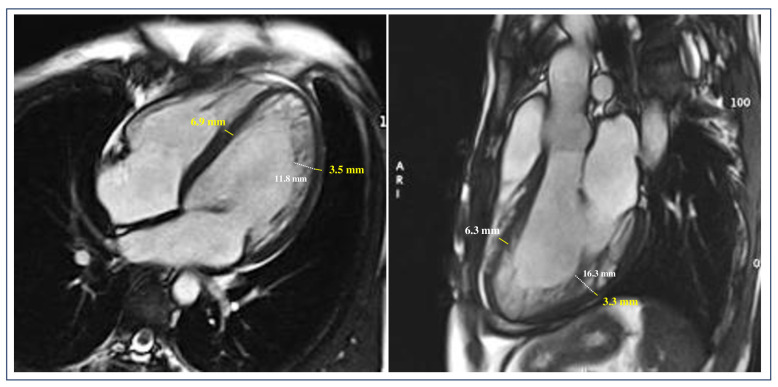
**A representative example of a patient with LVNC with LVEF reduction.** Diastolic frames of kinetic images in both four-chamber long axis and three-chamber long axis views showing a thinned compact layer with a thickness < 5 mm of the free-wall mid-ventricular segments. Note the free wall to septum asymmetry of thickness. Adapted from De Lazzari et al. [90]. LVNC = left ventricular non-compaction; LVEF = left ventricular ejection fraction.

**Figure 3:**
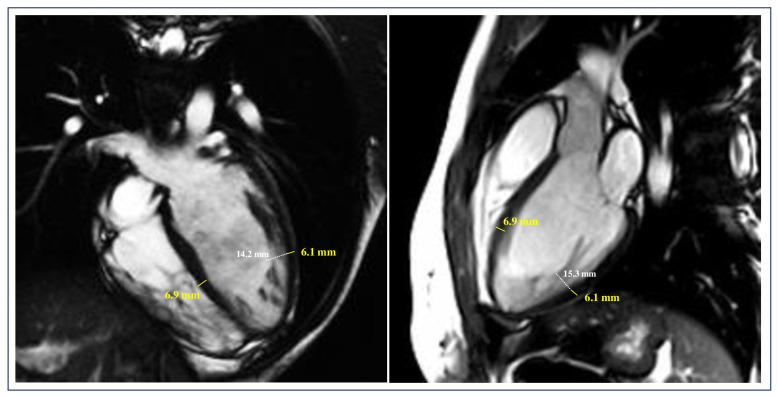
**A representative example of a patient with LVNC with preserved LVEF.** Diastolic frames of kinetic images in both four-chamber-long axis and three-chamber-long axis views showing a thickness of compact layer ≥ 5 mm. Adapted from De Lazzari et al. [90]. LVNC = left ventricular non-compaction; LVEF = left ventricular ejection fraction.

Using CMR imaging, the compact layer thickness in the LV Bull’s-eye segments was measured, and findings were compared between LVNCrEF patients and matched controls with LVNCpEF [90] (Figure 2 and Figure 3). The results confirmed that the failure to develop a sufficiently thick compact layer, rather than non-compacted trabeculation per se, was strongly linked to impaired systolic function in LVNC [90]. These observations align with earlier studies suggesting that isolated excessive trabeculation, in the absence of other markers of heart disease or congenital malformations, has limited clinical significance [74]. Indeed, large population studies have shown that about 20% of healthy individuals meet the Petersen LVNC criteria, with no association between high non-compaction to compaction (NC/C) ratios and systolic dysfunction or adverse clinical outcomes [74,81,87]. Earlier CMR findings have indicated that predictors of a poor clinical course in LVNC include LVEF < 50% and myocardial fibrosis or LGE. However, it remains unclear whether these studies included patients with true LVNC cardiomyopathy or those presenting excessive trabeculation due to other conditions, such as dilated cardiomyopathy. To avoid this potential diagnostic overlap, De Lazzari et al. exclusively included patients with “isolated” LVNC, excluding individuals with other cardiac conditions, such as LV dilation or LGE/myocardial fibrosis, despite meeting the Petersen criterion for excessive trabeculation [90]. This carefully selected cohort had a notably low incidence of clinical heart failure, ventricular tachycardia, and thromboembolic events, which are typically seen in more advanced cardiomyopathies. In the context of isolated LVNC, identifying a compact layer thickness cutoff related to reduced LVEF provided valuable diagnostic insights. More than two mid-ventricular segments with a compact layer under 5 mm were 100% sensitive for identifying isolated LVNC patients with reduced LVEF [90]. On the other hand, the absence of such findings predicted preserved LV function with 100% certainty [90]. Follow-up studies further confirmed that patients without a thinned compact layer maintained normal systolic function, whereas those with more than two affected segments exhibited worsening LV performance over time, as shown on serial echocardiography and CMR [90]. These results reinforce and expand upon previous echocardiographic studies, underscoring the potential pathological relevance of compact layer thinning. In one small case–control study, a compact layer under 5 mm in diastole, as measured by echocardiography, was more frequently observed in athletes with LVNCrEF than in those with preserved LVEF [91]. Among 36 athletes meeting the echocardiographic criteria for LVNC, three with LVEF below 50% had a compact layer less than 5 mm in systole and less than 4 mm in diastole [43]. The inability of non-compaction layer thickness or the NC/C ratio to predict LV systolic dysfunction supports the view that excessive trabeculation is not a unique marker of LVNC cardiomyopathy. Rather, it is a non-specific phenotypic trait seen in various diseases and conditions associated with increased cardiac load. This perspective is consistent with current embryological evidence, which challenges the outdated idea that the compact layer forms through trabecular compaction, suggesting instead that the compact and trabeculated layers develop independently via allometric growth [90].

## 11. Hypertrabeculation: What Is Hidden Behind?

Whether LVNC is a primary cardiomyopathy or a merely phenotypic trait is still a matter of debate. This distinction is crucial because of the clinical implication: in the first case, the goal is to identify patients with high-risk of cardiovascular events, while in the second, it might be unnecessary to require careful attention or closer follow-up. Recently, N. Miaris replied to De Lazzari et al.’s article [92]. In fact, it has been argued that there is a lack of clear evidence supporting the theory that human heart development involves the compaction of pre-existing trabeculations, and it is suggested that the trabeculated and compact myocardial layers develop independently, rather than through a failure of compaction that results in distinct compact and non-compact layers of the LV wall [84]. The author emphasized that these reasons has led the ESC guidelines to dismiss the term “LV non-compaction” in favor of “hypertrabeculation”, viewing it as a phenotypic trait associated with other cardiomyopathies or found in isolation, particularly in cases with normal LVEF and favorable prognosis, rather than as a distinct cardiomyopathy. Notably, the term ‘non-dilated LV cardiomyopathy’ (NDLVC) has been introduced to describe cases characterized by preserved LV size with scarring or systolic dysfunction [75]. According to the letter, cases featuring a non-dilated LV cavity, systolic dysfunction, and hypertrabeculation that meet any previously established imaging criteria should be now reclassified as NDLVC [92]. This is based on the understanding that the presence of hypertrabeculation does not alter patient management, and prognosis is more strongly influenced by the underlying condition rather than by the trabeculations themselves.

By contrast, De Lazzari et al. affirmed that while the appropriateness of the term “left ventricular non-compaction” may be open to debate, it is undeniable that dismissing the existence of this cardiomyopathy based solely on the arbitrary consensus of an ESC task force, without robust scientific evidence, is not acceptable [93]. Instead, they added that their manuscript presents the possibility of a paradigm shift in how this cardiomyopathy is categorized: moving away from the “old concept” of a congenital compaction defect of the LV myocardium, toward a modern perspective that recognizes the embryological underdevelopment of the compact layer—independent of the trabeculated layer’s growth—as a disease-specific pathophysiologic mechanism that impairs LV function [93]. Growing evidence demonstrated that the compact layer and trabeculated layers develop independently of each other. Accordingly, hypertrabeculation is a non-specific trait reported in other diseases and in some overloading conditions and does not represent a distinctive morpho-functional hallmark of LVNC. Moreover, in agreement with recent studies, hypertrabeculation meeting current LVNC diagnostic criteria has no significant association with worse prognosis. Results found by Andreini et al. were in keeping with Amzulescu’s experience [83,87], which showed that cardiovascular outcomes of patients with DCM were not influenced by the degree of trabeculations. Similarly, in the MESA trial, the authors reported that LV hypertrabeculation extensions in asymptomatic patients was not associated neither with LV dilatation nor systolic dysfunction during a 10-year follow-up [81]. In adults diagnosed with hypertrophic or dilated cardiomyopathy where excessive trabeculation is also present, the extent of ventricular trabeculation has not been demonstrated to alter management nor prognosis.

These characteristics make LVNC a two-faced Janus: on one hand, it appears to be secondary to underdevelopment of the non-compacted layer that manifests early in childhood, while on the other hand, it seems to be an epiphenomenon related to adaptive stimuli such as pressure or volume overload conditions.

This distinction is crucial for improving diagnosis, prognostic assessments, and treatment strategies. Although LVNC shares prognostic factors with conditions resembling DCM, the full understanding of LVNC’s natural history is complicated by confounding variables during patient selection for studies. Establishing LVNC as a distinct nosological entity with a peculiar disease-specific mechanism will promote the development of more standardized treatment protocols, improving patient care.

## 12. Future Perspectives

The present review emphasizes the concept of making a diagnosis by shifting the perspective on LVNC, focusing on the thinning of the compact layer rather than the thickening of the non-compacted layer. Which patient has an LVNC cardiomyopathy, and in which patient is LVNC merely an epiphenomenon? To address this issue, De Lazzari et al. elaborated a hypothesis-generating study based on asymmetry in the thickness between the free wall and the septum [90]. This discrepancy could be a distinct morphologic feature that may further characterize the isolated LVNC cardiomyopathy phenotype and aid in discriminating it from DCM with superimposed excessive trabeculation [90]. In fact, DCM is characterized by a harmonic thickness of both the free and lateral wall based on the “eccentric hypertrophy” concept in which the thickness of both septum and free wall is symmetrical. However, larger multicenter studies are warranted to confirm this hypothesis. Data on long-term outcomes of LVNC cardiomyopathy excluding patients with DCM and secondary hypertrabeculation may be desirable. The role of the scar in LVNC, both in terms of diagnosis and arrhythmic risk stratification, still remains an issue yet to be fully elucidated. Multimodal artificial intelligence (AI) is a novel technological tool that enables the integration of information with the aim of stratifying arrhythmic risk [94]. Despite clinical application obstacles, AI has the potential to offer opportunities to expand knowledge in the cardiomyopathy scenario. Particularly among similar variants of cardiomyopathies such as LVNC and DCM, computational models will provide imaging-related elements to differentiate morphological key features [94]. Further studies on AI prediction models are warranted for a widespread clinical adoption.

## 13. Conclusions

Excessive trabeculation, when not accompanied by thinning of the compact layer, appears as a “phenotypic trait” rather than a “cardiomyopathic morphological marker”, lacking clinical and prognostic significance. Future prospective research should focus on the clinical and prognostic relevance of compact layer thinning rather than excessive trabeculation, which could significantly influence the management of patients with LVNC.

## Figures and Tables

**Table 1:** LVNC most common diagnostic criteria.

	Jenni et al. [7]	Petersen et al. [60]	Jacquier et al. [61]	Stacey et al. [62]	Captur et al. [63]
**Method**	TE	CMR	CMR	CMR	CMR
**Overall population**	NC (n = 34) No control	NC (n = 7) Control (n = 170)	NC (n = 16) Control (n = 48)	NC (n = 122) No control	NC (n = 30) Control (n = 105)
**Cardiac phase**	End-systole	End-diastole	End-diastole	End-systole	End-diastole
**Cut-off**	NC/C > 2	NC/C > 2.3	Trabecular mass > 20%	NC/C > 2	Fractal dimension > 1.3

Legend: TE = transthoracic echocardiography; CMR = cardiac magnetic resonance; NC = non-compacted (layer); C = compacted (layer); n = number of patients.

## Data Availability

Data are available on request from the authors.
